# Modular network mechanism of CCN1-associated resistance to HSV-1-derived oncolytic immunovirotherapies for glioblastomas

**DOI:** 10.1038/s41598-021-90718-1

**Published:** 2021-05-27

**Authors:** Dileep D. Monie, Cristina Correia, Cheng Zhang, Choong Yong Ung, Richard G. Vile, Hu Li

**Affiliations:** 1 Medical Scientist Training Program, Mayo Clinic College of Medicine and Science, Mayo Clinic, 200 First Street SW, Rochester, MN 55905 USA; 2 Department of Immunology, Mayo Clinic College of Medicine and Science, Mayo Clinic, 200 First Street SW, Rochester, MN 55905 USA; 3 Center for Regenerative Medicine, Mayo Clinic College of Medicine and Science, Mayo Clinic, 200 First Street SW, Rochester, MN 55905 USA; 4 Department of Molecular Pharmacology and Experimental Therapeutics, Mayo Clinic College of Medicine and Science, Mayo Clinic, 200 First Street SW, Rochester, MN 55905 USA; 5 Center for Individualized Medicine, Mayo Clinic College of Medicine and Science, Mayo Clinic, 200 First Street SW, Rochester, MN 55905 USA

**Keywords:** Cellular signalling networks, Systems biology, Tumour immunology

## Abstract

Glioblastomas (GBMs) are the most common and lethal primary brain malignancy in adults. Oncolytic virus (OV) immunotherapies selectively kill GBM cells in a manner that elicits antitumor immunity. Cellular communication network factor 1 (CCN1), a protein found in most GBM microenvironments, expression predicts resistance to OVs, particularly herpes simplex virus type 1 (HSV-1). This study aims to understand how extracellular CCN1 alters the GBM intracellular state to confer OV resistance. Protein–protein interaction network information flow analyses of LN229 human GBM transcriptomes identified 39 novel nodes and 12 binary edges dominating flow in CCN1^high^ cells versus controls. Virus response programs, notably against HSV-1, and cytokine-mediated signaling pathways are highly enriched. Our results suggest that CCN1^high^ states exploit IDH1 and TP53, and increase dependency on RPL6, HUWE1, and COPS5. To validate, we reproduce our findings in 65 other GBM cell line (CCLE) and 174 clinical GBM patient sample (TCGA) datasets. We conclude through our generalized network modeling and system level analysis that CCN1 signals via several innate immune pathways in GBM to inhibit HSV-1 OVs before transduction. Interventions disrupting this network may overcome immunovirotherapy resistance.

## Introduction

Glioblastomas (GBMs) are particularly aggressive primary brain tumors that are relatively common in adults^1^. The disease comes with a dismal prognosis, often with expected survival just over a year when treated with surgery, chemotherapy, and radiation^2^. Immunotherapies such as checkpoint inhibitors and chimeric antigen receptor (CAR) T cells are emerging as a fourth arm in the treatment arsenal against GBM. Another class of immunotherapy now in clinical trials for GBM is oncolytic viruses (OVs)^3^. Unlike CAR-T cell immunotherapies, OVs have been FDA approved for a solid tumor: an engineered herpes simplex virus type 1 (HSV-1) for melanoma^4^. More recently, an engineered HSV-1 OV has demonstrated response in pediatric high-grade glioma with evidence of an immunological mechanism^5^. OVs debulk by targeting tumors with high specificity and may offer immune-mediated protection against tumor recurrence. The most effective OVs home in on local and metastatic cancer cells, lysing them and releasing tumor-associated antigens in the context of proinflammatory signals that elicit antitumor immunity^6^. This effectiveness, however, varies greatly depending on the cell state as defined by its transcriptomic profile.

GBM cell states are dynamic and influenced by several factors^7^, including the composition of the extracellular matrix (ECM). Cellular communication network factor 1 (CCN1) is a protein found in the ECM of the majority of GBMs and is predictive of resistance to OVs, particularly those derived from HSV-1^8^. CCN1^high^ expressing GBMs also confer worse progression-free and overall survival prognoses^9^. A prior study found that CCN1 binds and activates cell surface integrin α6β1, promoting an antiviral and protumoral state via the secretion of interferon-α^8^. Microarray-based heatmap and pathway analysis from this study showed type I interferon stimulated gene (ISG) expression and associated signaling in LN229 GBM cells. Haseley *et al*. conclude that CCN1 is a marker of HSV-1 OV resistance and propose blocking CCN1–integrin α6 interactions to restore permissiveness to this therapy.

Because the ECM is difficult to disrupt pharmacologically and CCN1 is stoichiometrically abundant, we asked if there are downstream protein–protein interactions (PPIs) essential for the observed CCN1^high^ GBM phenotype. In this study, we constructed global PPI networks from the published datasets using process-guided flow algorithms^10^ and then analyzed information flows to derive a prioritized subnetwork, as well as identify high impact genes, network routers, key targets, and CCN1-specific edges. We elucidated novel pathways, proteins, and interactions critical to CCN1^high^ GBM phenotype that are potentially druggable and can guide the engineering of precision OVs.

## Results

Our systems biology approach to identify opportunities for improved HSV-1 OV design consisted of network and motif modeling, overrepresentation analysis, assessment of gene dependencies, and confirmation of gene expression in clinical tissue samples. Publicly available microarray data on CCN1-induced and control samples^8^ were used as the starting point for our investigation. Our NetDecoder analysis yielded several high impact genes, notable for their differential edge flows, organized in a prioritized subnetwork. Our results indicate 39 nodes that may influence susceptibility of CCN1-expressing GBM to OV. Of these, a router (IKBKE) and a sink (YBX1) have been implicated in GBM pathogenesis. Furthermore, category enrichment suggests that measles virus may be more effective in these types of tumors.

### GBM CCN1 context-specific network model

**Figure 1:**
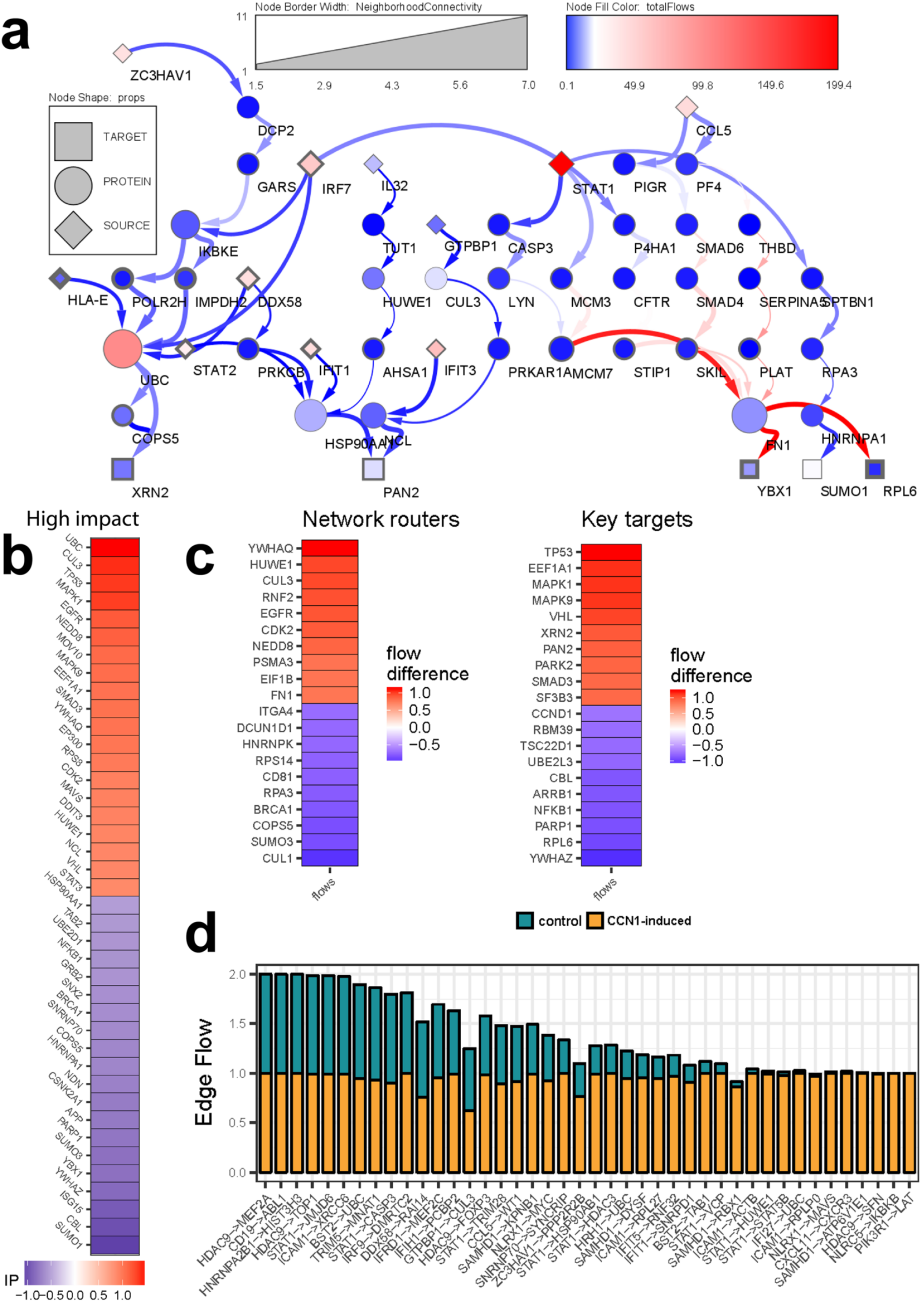
Prioritized flow subnetwork in glioblastoma. (**a**) Prioritized protein–protein interaction subnetwork. These interactions are represented by nodes (genes) and edges (interactions) with higher (red) and lower (blue) differential flows under CCN1-induced phenotype *versus* the uninduced control phenotype. Nodes consist of sources (diamonds), routers (circles), and sinks or targets (squares). Sources are previously published differentially expressed genes CCN1 induced biological states. (**b**) High impact genes experience significant shifts in regulation due to the number and directionality of interacting partners across phenotypes. (**c**) Flow differences. Heatmap showing node flow differences across uninduced control and CCN1-induced LN229 human GBM cells (n = 3 replicates) for top 20 network routers and key target genes showing high node flow difference (red) and low node flow difference (blue) in CCN1-induced GBM cells. (**d**) Key edges. Total edge flow profiles in uninduced control and CCN1-induced LN229 human GBM cells for edges with higher flows in CCN1-induced than in control subnetworks.

We used previously identified differentially expressed genes^8^ as the starting point for our PPI networks to derive the prioritized context-specific GBM subnetwork shown in Fig. 1. By comparing the CCN1^low^ and CCN1^high^ PPI networks we derive a subnetwork that captures key differences between the two biological states. Our NetDecoder analysis yielded 50 genes in a prioritized subnetwork of high impact genes, notable for their differential edge flows. Of these, 11 genes are flow sources that were previously reported as differentially expressed between CCN1 induced and control cells. Within our prioritized network, the source genes collaborate via 34 network routers to signal to 5 downstream targets (XRN2, PAN2, YBX1, SUMO1, and RPL6) (Fig. 1a and Supplementary Table S1). Of these, IKBKE (inhibitor of nuclear factor kappa-B kinase subunit epsilon) and YBX1 (Y box binding protein 1) have been previously implicated in glioblastoma pathogenesis and metabolic targeting of virotherapy^11,12^. IKBKE was identified as a 4-edge router showing a lower flow in CCN1 induced cells, this gene has been described to have an impact on and glioblastoma resistance to apoptosis and engaging NF-κB activation leading to antiviral program^11^. Our methodology allows us to dissect novel subnetwork nodes and expose other targets for biological validation and possible therapeutic intervention. FN1 (fibronectin 1), a 6-edge router with higher flow in CCN1^high^, is one of the most upregulated genes in gliomas^13^. In Fig. 1b, we elucidate several high impact (IP) genes, including UBC, a 7-degree hub within the CCN1 subnetwork. UBC has been described to be involved in the viral and replication control^14^ and its interaction with the proteasome facilitates HSV entry^15^ which leads us to postulate that this ubiquitin proteasome system confers OV resistance to CCN1-expressing glioblastomas.

Figure 1c captures the flow difference across biological states and we observe that the flow through TP53 is greatly impacted. The *TP53* gene encodes tumor suppressor protein p53, a transcription factor in the p53-ARF-MDM2 pathway that is dysfunctional in 84% of GBM cases and 94% of GBM cell lines, including LN229^16^. In the context of CCN1 the cell signaling is decreased for NFKB. Additionally, we find that several key edges that dominate in or are exclusive to CCN1^high^ cells (Fig. 1d). For example, IKBKE interaction with the pattern recognition receptor NLRC5 forms a key edge, further suggesting that this may play an important role in CCN1-mediated immunovirotherapy resistance. This is consistent with decreased flows to NFKB1 because NLRC5 drives transcription of the NF-κB inhibitor *IKBKB*^17^. Another edge unique to CCN1^high^ GBM is PIK3R1 phosphorylation of LAT, critical for initiation of immune cell activation^18^.

### Overrepresentation analysis

**Figure 2:**
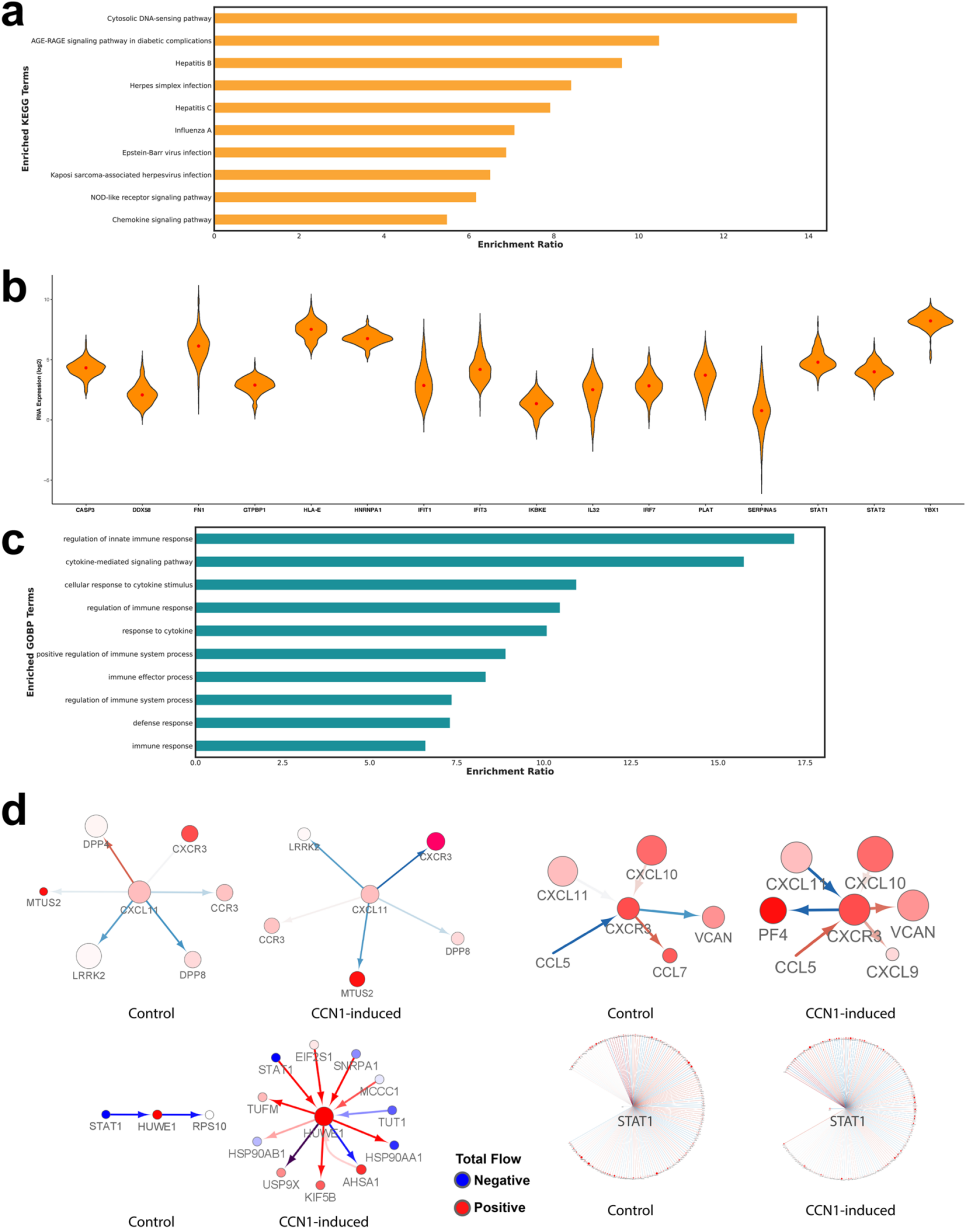
Glioblastoma enriched gene categories. (**a**) Overrepresentation enrichment KEGG analysis was performed using WebGestalt for the genes (n = 50) in CCN1 prioritized subnetwork. As expected, several genes used as flow sources in our network were reported within multiple enriched categories. (**b**) Violin plots showing the mRNA expression of HSV-1 (KEGG pathway: hsa05168) enriched genes in TCGA GBM patients (n = 174). (**c**) Overrepresentation enrichment GO Biological Process analysis was performed using WebGestalt for the genes (n = 21) involved in edges that dominate the network flow in CCN1-induced LN229 cells versus control (see Fig. 1d and Supplementary Fig. S1). We find that these edges comprise several cytokine signaling and immune response pathways. (**d**) CCN1-specific edge impact motifs for control and CCN1-induced. Rewiring is observed for CXCL11:CXCR3 and STAT1:HUWE1 pairs. Node degree and edge flows are drastically increased for HUWE1 in the context of CCN1 overexpression.

Next, to explore altered pathways identified in our biological subnetwork we performed a Kyoto Encyclopedia of Genes and Genomes (KEGG)^19^ pathway overrepresentation analysis (ORA) (Fig. 2a and Supplementary Fig. S1) and focused on the newly identified nodes in our prioritized subnetwork. Our results confirm that the presence of CCN1 alone primes GBM cells to resist HSV-1 (-log_10_FDR = 3.67; enrichment ratio = 8.42). This analysis also suggests that other viruses, such as adenovirus, may be a more effective starting point for constructing an OV for CCN1^high^ GBM patients. To confirm that these key genes are expressed in GBM, we next explored The Cancer Genome Atlas (TCGA; https://www.cancer.gov/tcga) GBM datasets^20^ (n = 174) to detect that most of the genes are present (Fig. 2b). To further interrogate the CCN1^high^ edges we selected the 12 most dominant edges from Fig. 1D and performed ORA for gene ontology biological processes (GOBP) to reflect an impact in cytokine signaling (Fig. 2c). We found that the cytokine-mediated signaling pathway (-log_10_FDR = 10.37; enrichment ratio = 15.76) and the cellular response to cytokine stimulus (-log_10_FDR = 8.50; enrichment ratio = 10.95) were highly enriched when mapped to these nodes. From these top GOBP terms for cytokine signaling, we selected the CXCL11:CXCR3 and HUWE1:STAT1 interaction edge pairs (Fig. 2d top panel) to inspect their network motifs in CCN1 control and CCN1-induced biological states. The CXCL11:CXCR3 interaction is present in both states but increased the flow through its receptor CXCR3. This suggests a mechanism for microglial activation and leukocyte recruitment^21^. For the HUWE1:STAT1 interaction (Fig. 2d bottom panel) we observed that HUWE1 (very high flow node) suppresses N-Myc-DLL3, which may be responsible for promoting neurogenesis. Genetic and epigenetic inactivation of HUWE1 has been shown to promote tumorigenesis in GBM^22^. Modulation of HUWE1 is pharmacologically feasible and a promising therapeutic strategy^23^. The number of biological partners (node degree) is dramatically altered across states, which suggests rerouting upon CCN1 overexpression.

### Motif analyses in CCN1 induced states

**Figure 3:**
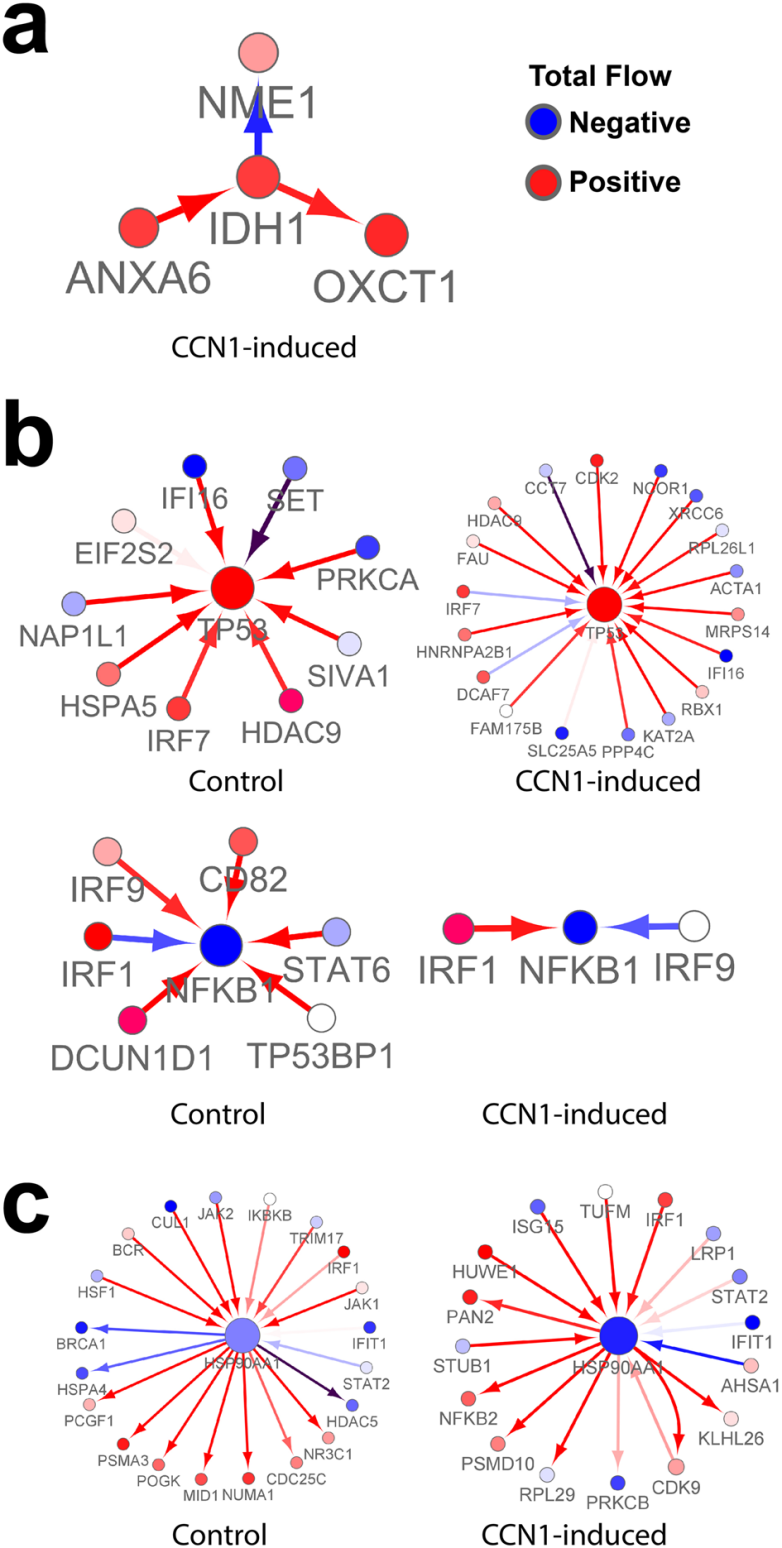
Motif analysis of key genes. (**a**) GBM prognostic marker: IDH1 (only appears in CCN1-induced global network). (**b**) Key Targets: TP53 (increased connectivity in CCN1-induced) and NFKB1 (decreased connectivity in CCN1-induced). (**c**) High impact genes: HSP90AA1. Some rewiring is observed in this autophagy regulator.

Molecular determinants in GBM are *IDH1* mutation status and *MGMT* promoter methylation, which impact disease outcome and therapy strategy^24^. To uncover the IDH1 and 2 we inspected the network neighborhood motifs in our CCN1 induced network (Fig. 3a). LN229 is an *IDH1* wild type glioblastoma and *TP53* mutant cell line derived from the right frontal parieto-occipital cortex of a 60-year old female. IDH1 is a limiting enzymatic factor in cellular energy metabolism and, consequently, IDH1 mutations are prognostic of better overall survival in GBM^25^. IDH1 was only detected in our CCN1-induced state network where it had significant flow, suggesting that IDH1 has a functional role in this state. Our motif analysis reveals its key interactions involve ANXA6, OXCT1, and NME1 that contribute for the rewiring of this state. Interestingly there is some supporting literature evidence linking these genes to GBM pathophysiology^26–28^. Using Cancer Cell Line Encyclopedia (CCLE) data^29^, CCN1 expression levels are weakly correlated with IDH1 (ρ =  − 0.273; *p* value = 0.027) (Supplementary Fig. S2a).

Because we have *TP53* mutant cell lines and *TP53* mutations have been identified in GBM^20^, we inspected its motif and observed that CCN1 increased the interaction with TP53 (Fig. 3b right panel). NFKB1 a central immune regulator was previously detected in our analysis (Fig. 3b left panel and Fig. 1c). We observe that CCN1 induced state as decreased edge flow and altered connectivity to the genes and mostly dependent on IRF1. Autophagy and chaperone interaction network regulator HSP90AA1, a critical 5-degree hub node, may be targeted to improve OV efficacy in CCN1-expressing glioblastomas (Fig. 3c)^30^**.** Such an approach would be anticipated to trigger type II cell death via AKT/mTOR inactivation in this context^31^.

### Gene dependency analysis

**Figure 4:**
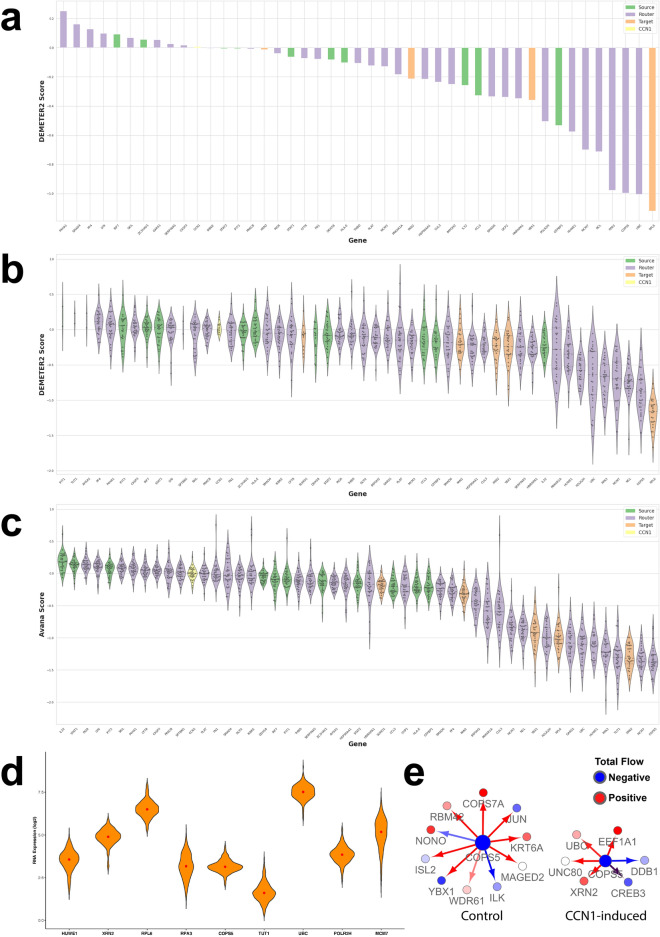
Gene dependencies in glioblastoma prioritized subnetwork. (**a**) Waterfall plot showing DEMETER2 scores for the LN229 human GBM cell line for genes identified in the CCN1 NetDecoder subnetwork. (**b**) Violin plot showing RNAi gene dependencies (DEMETER2 scores) for nodes in our GBM NetDecoder analysis. Depicted are GBM cell lines (range: n = 2–31). (**c**) Violin plot showing CRISPR gene dependencies (Avana scores) for nodes in our GBM NetDecoder analysis. Depicted are the GBM cell lines (range: n = 28–33). (**d**) Violin plot showing TCGA RNA expression for selected genes with low gene dependency score in GBM patients (n = 174). Colors assigned to network source, router, target genes and CCN1 are green, purple, orange, yellow, respectively. (**e**) Network router motif: COPS5. Connections decrease when CCN1 is overexpressed.

Since HSV-1 OV shows significant promise in some GBM, we wanted to explore how we can improve its efficacy in refractory GBM cells. Given that CCN1 is a critical factor for HSV-1 resistance, we investigated the dependencies of the genes in our prioritized subnetwork using the gene dependency datasets DEMETER2 (RNAi) and Avana (CRISPR) (Fig. 4a–c)^32,33^ to further dissect GBM cancer vulnerabilities. Here, we focused on CCN1 network dissected genes and inspected the LN229 cell line (Fig. 4a). We then expanded to all available GBM cell lines (range: n = 2–35) (Fig. 4b, c and Supplementary Table S2) to observe agreement of the genes showing dependencies in the GBM for RPL6, HUWE1, and COPS5. In Fig. 4d, we inspected the TCGA expression levels of genes that contribute to gene dependency in GBM, defined as having an Avana score less than -1.0. In Fig. 4e, we zoomed into COPS5—a network router and vulnerability gene—to find reduced connectivity and a rerouting through UBC and EEF1A1. Target XRN2, an exoribonuclease, suggests that our prioritized subnetwork confers some of its phenotype-defining effects through alteration in the DNA damage response, known to be mediated through Ku70 in LN229 and other GBM cells^34^.

### Validation of findings in CCLE and TCGA datasets

**Figure 5:**
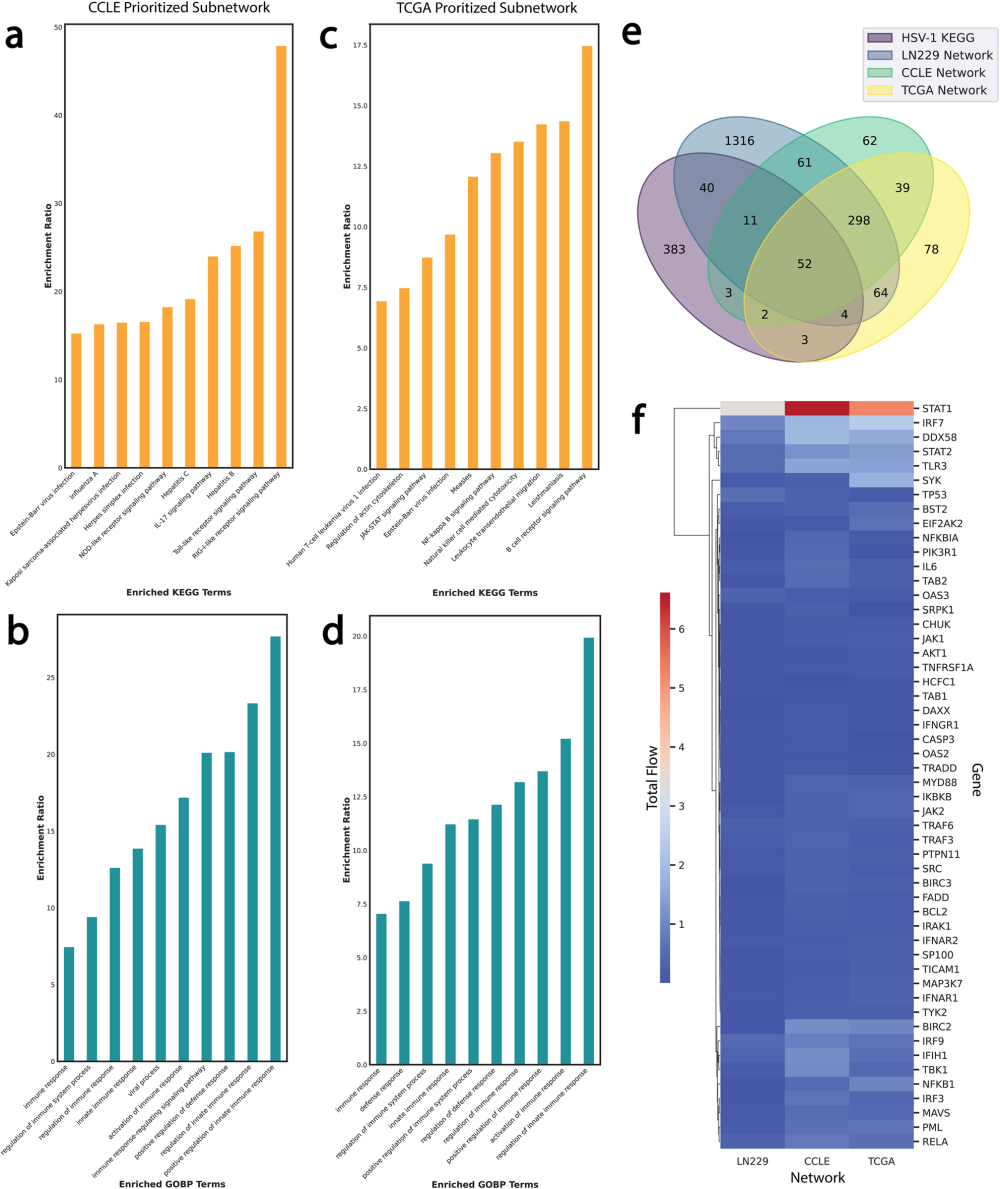
CCN1^high^-specific networks in CCLE and TCGA GBMs. WebGestalt ORA of (**a**) KEGG pathways and (**b**) GOBP terms in the CCLE GBM prioritized subnetwork nodes (n = 30) generated using the source genes (n = 57) most differentially expressed in CCN1^high^ LN229 cells. WebGestalt ORA of (**c**) KEGG pathways and (**d**) GOBP terms in TCGA GBM prioritized subnetwork nodes (n = 43) generated using the source genes (n = 57) most differentially expressed in CCN1^high^ LN229 cells. (**e**) Venn diagram identifies network genes across LN229 (n = 1,846), CCLE (n = 528), and TCGA (n = 540) datasets found in the HSV-1 KEGG pathway (n = 498). (**f**) Euclidean average cluster analysis heatmap of the total flow through the 4-way intersecting genes (n = 52).

To validate our findings, we devised a two-fold validation approach by using CCLE and patient TCGA GBM RNA-seq datasets. First, we used CCN1 median expression level for each dataset to stratify cell lines into CCN1^high^ and CCN1^low^ bins (Supplementary Fig. S2b). This approach is similar to the strategy depicted for LN229 inducible CCN1 overexpressing system. Then we generated context specific networks in GBM cell lines. The same analysis was performed with TCGA GBM data. By using broader datasets we can better capture the spectrum of CCN1-driven networks across diverse biological contexts. Next our CCLE- and TCGA-context specific networks were subjected to NetDecoder analysis. Figure 5a shows herpes simplex infection in the top overrepresented KEGG pathways (-log_10_FDR = 6.87; enrichment ratio = 16.63) in the CCN1^high^-specific prioritized subnetwork of CCLE GBMs (n = 30) generated using the source genes (n = 57) most differentially expressed in CCN1^high^ LN229 cells (Supplementary Fig. S3a). Two other infection pathways triggered by similar Baltimore class I dsDNA viruses—Epstein Barr virus (EBV) and Kaposi’s sarcoma-associated herpesvirus (KSHV)—are also overrepresented. Overrepresented GOBP terms in this subnetwork all involve the immune response (Fig. 5b). A similar theme is seen in the TCGA GBM prioritized subnetwork nodes (n = 43) generated using the same source genes (Fig. 5c, d; Supplementary Fig. S3b). Even though individual node overlap (Supplementary Fig. S3c) is unremarkable, likely reflecting the heterogeneity of disease captured by broader CCLE and TCGA data, the overlap between the three prioritized subnetworks reproduces the same pathways. This is consistent with our initial findings in LN229 GBM cells and allows generalization to other GBM cell lines in vitro and patients’ tumors in situ.

We then identified the genes common across LN229 (n = 1,846), CCLE (n = 528), and TCGA (n = 540) global networks that are also found in the HSV-1 infection KEGG pathway^19^ (hsa05168; n = 498), diagrammed in Fig. 5e. Cluster analysis of the total flow through the 4-way intersecting genes (n = 52) shown in Fig. 5f suggests that STAT1, IRF7, and DDX58 are the HSV-1 infection response genes with the highest flows and contribute to the modulation of CCN1^high^ networks. These genes are critical components of the innate immune response to viruses^35^.

### Strategies to increase HSV-1 OV efficacy in CCN1-expression GBM

Inspection of Drugbank (https://go.drugbank.com/) identified Lyn kinase, a low flow node in our prioritized subnetwork, as a potential targetable gene and previous studies in GBM identified Lyn kinase activity is significantly elevated in the glioblastoma biopsy samples^36^. A clinical trial (NCT01234740) using the dual BCR-Abl/Lyn tyrosine kinase inhibitor bafetinib did not yield clinically significant results due to poor neuropharmocokinetics so better agents or delivery systems may be required^37^. More recently E3 ligase HUWE1 inhibition was described as a therapeutic strategy to target MYC in multiple myeloma^38^ and small drugs are under development^39,40^.

Given that CCN1-expressing glioblastomas activate an antiviral program that particularly resists HSV-1 OV, we examined how to improve the HSV-1 chassis. Studies have shown that HSV-1 vectors can carry short-hairpin RNA (shRNA) or microRNA (miRNA) payloads and these can silence target genes in the host cells^41,42^. The miRNA strategy has been demonstrated to be effective at blocking interferon responses in vivo to enhance efficacy against nervous system-derived tumor cells^43^. Therefore, this approach is particularly attractive for perturbing critical nodes in our network analysis since they exist in GBM cells targeted by, but resistant to, HSV-1 OV. If pretreatment resistance proves to be insurmountable, these shRNA and miRNA vectors could also be delivered using another viral vector to prime the cell state to be permissive to HSV-1 in a tandem OV therapeutic approach^44^.

## Discussion

In this work, we aimed to model and classify GBM responses to OVs. We utilized a computational systems biology approach based on network analysis of transcriptomics data, gene dependencies datasets (DEMETER2 and Avana), and TGCA and CCLE expression in GBM. Our novel genome-wide flow-based systems strategy unravels protein–protein interactions that are important in GBM development and progression. Most importantly, our network approach prompts us to uncover the role of genes that importance derives from the number of interactions and associated partners. By better understanding these networks, targeted therapies can be developed to improve outcomes for patients.

The results of this analysis suggest several novel potential therapeutic targets for OV of CCN1^high^ GBM. The presence of validated targets, IKBKE and YBX1, in our network indicate that flow differences can be served as indicators to guide precision engineering of OV. Overrepresentation analysis elucidated the role of CCN1 on OV therapies and highlighted the immune contribution through the CXL11:CXCR3 and NFKB1 axis. We further investigated the impact of known GBM molecular determinants in our full network and inspected motifs to find that the CCN1-induced state is more IDH1 flow dependent. Because *TP53* mutation is a common event in primary glioblastoma and was both a high impact and key target gene in our network analysis, we inspected its network neighborhood to find a dramatic rewiring and increase of biological partners that contribute to CCN1^high^ state. These new interactions, combined with the loss of other interactions, likely inhibit the tumor suppressor activity of TP53 and thus may play a role in GBM progression.

To uncover additional GBM vulnerabilities we focused on hidden genes identified with our network analysis and utilized gene dependency scores to find that RPL6, HUWE1, and COPS5 contribute for GBM dependencies. Inspection of TCGA GBM dataset confirmed that these genes are expressed in GBM. Diving into our context-specific networks to retrieve potential targetable pathways and individual genes revealed that HUWE1 is a targetable gene and consider further inspection on its role and regulation mechanisms in GBM.

To validate our LN229 findings we expanded our analysis to in vitro (CCLE) and in vivo datasets (TCGA). This analysis confirmed and further elucidated context-specific networks that captured biologically relevant disease heterogeneity and revealed key regulators in the HSV-1 pathway—specifically STAT1, IRF7, and DDX58—generalizable to dsDNA-based oncolytic immunovirotherapy responses in GBM patients.

One limitation of this work is that the source data used to generate our context-specific subnetwork is based on microarrays of mRNA, which may not accurately represent functional proteins that interact in the predicted networks. Additionally, the mRNA samples were prepared from cell lines, which lack the complexity of an in vivo GBM tumor milieu (e.g. stromal, immune, and heterogeneous cancer cells). The use of this disease data was mainly driven by the availability of data but we believe that a similar benchmarking study on GBM patient derived xenograft (PDX) models will be amenable to such studies and we anticipate that flow differences will help predict tumor responses, adverse events, and suggest effective neoadjuvants (e.g. IKK inhibitors) and yield targetable genes. Our in silico findings across diverse datasets generate hypotheses that are testable in the wet lab, likely by perturbing genes identified with network differential flows (e.g. IKBKE and YBX1) that may alter OV susceptibility, suggesting biological significance.

Overall, our strategy and results may aid the design of next generation OVs. This may take the form of OVs armed with synthetic gene circuits, as well as OV cocktails with multiple tropisms and physiological effects. Desired outcomes are not only overcoming resistance, but also improved oncolysis, stimulation of antitumor immunity, and even promotion of tissue regeneration. Ultimately, the initial OV-mediated inflammation in the brain will need to be resolved and neuroimmune homeostasis restored.

## Methods

### Datasets and preprocessing

Raw microarray data (*.cel files) Affymetrix Microarray dataset (Human Genome U133 Plus 2.0) was retrieved from GEO accession GSE29384 (https://www.ncbi.nlm.nih.gov/geo/), and then processed with Affy^45^ and Limma^46^ R packages. CCLE RNA-seq data was downloaded (https://portals.broadinstitute.org/ccle/data) in March 2021 and central nervous systems cell lines were selected (n = 66). RNA normalized expression of TCGA GBM patients (n = 174) was retrieved from the NCI Genomic Data Commons Data Portal (https://portal.gdc.cancer.gov) in January 2021. GDC-RNAseq tool (https://github.com/cpreid2/gdc-rnaseq-tool) was used to compile RNA sequence (RNA-seq) data. R (version 1.3) ggplot2 was used for the RNA-seq analysis and fragments per kilobase of transcript per million mapped reads with upper quartile normalization (FPKM-UQ) was used for plotting and NetDecoder analysis.

### Network analysis

We used NetDecoder^10^ (https://github.com/HuLiLab/NetDecoder) to elucidate protein–protein interaction networks in publicly available microarray data from LN229 human GBM cells treated with HSV-1 OV. These cells have tetracycline-inducible expression of the OV-inhibitory ECM protein CCN1. Differentially expressed genes from Haseley *et al*.^8^ were used as sources in NetDecoder, which was run using default parameters. Co-expression networks were derived from transcriptome of LN229 CCN1-induced and -uninduced states. Our network analyses prioritize human genes that are differentially expressed (sources) between CCN1-induced and -uninduced control cell phenotypes.

For the CCLE dataset, we selected all central nervous systems (CNS) cell lines (n = 66). CCN1 expression was assessed and median expression levels were used to stratify CCLE cell lines as CCN1^high^ or CCN1^low^. Differential expression analysis and normalization was performed with the DESeq2 R package^47^. Co-expression context specific networks were derived from normalized counts of CCLE CNS for CCN1^high^ and CCN1^low^ states. Network analysis was performed using the prioritized human genes across LN229 conditions as sources in a CCLE co-expression network. Size of functional neighborhood, SNF = 0.95, ratioThreshold = 5, and corThreshold = 0.5 were used as the default parameters for NetDecoder runs.

For TCGA network analysis, first we used TCGA normalized RNA-seq counts to stratify all GBM patients (n = 174) according to median tumor CCN1 expression. Next, context specific co-expression networks were generated for CCN1^high^ and CCN1^low^ GBM states. NetDecoder analysis was then performed using TCGA GBM specific co-expression networks and prioritized human genes across LN229 conditions as sources.

### Gene dependency analysis

Gene dependencies from two independent datasets were pulled: i) Combined RNAi (Broad, Novartis, Marcotte) gene dependencies (DEMETER2 v6) and ii) CRISPR Avana gene dependencies (20Q4 v2) from the Cancer Dependency Map (DepMap) Portal (https://depmap.org/portal/). Selection and analysis of these data were performed using Python version 3.6.9 in a Jupyter Notebook environment. Visualization was performed using Matplotlib version 3.3.3 (https://matplotlib.org/) and Seaborn version 0.11.1 (https://seaborn.pydata.org/).

### Enrichment analysis

WEB-based GEne SeT AnaLysis Toolkit (WebGestalt; http://www.webgestalt.org/) with KEGG and Gene Ontology (GO) functional databases was used for pathway enrichment analysis^48^. Input gene symbol lists were analyzed for overrepresentation against the *Homo sapiens* genome protein-coding reference set.

### Custom code

Analysis code, networks, and raw data are available at: https://github.com/HuLiLab/GBM_CCN1.

## Supplementary Information

Supplementary Information 1.
