# Supplementary material for: Modular network mechanism of CCN1-associated resistance to HSV-1-derived oncolytic immunovirotherapies for glioblastomas
Source: Sci Rep. 2021 May 27;11:11198. doi: 10.1038/s41598-021-90718-1 (PMC8159930; doi:10.1038/s41598-021-90718-1)
Supplement: Supplementary file 1 — Supplementary Information 1. [file 41598_2021_90718_MOESM1_ESM.pdf]

Title

Modular network mechanism of CCN1-associated resistance to HSV-1-derived oncolytic immunovirotherapies for glioblastomas

Authors

Dileep D. Monie[1,2,3], Cristina Correia, Ph.D.[4,5], Cheng Zhang, Ph.D.[4,5], Choong Yong Ung, Ph.D.[4,5], Richard G. Vile, Ph.D.[2], Hu Li, Ph.D.[4,5]*

Affiliations

[1]Medical Scientist Training Program, [2]Department of Immunology, [3]Center for Regenerative Medicine, [4]Department of Molecular Pharmacology and Experimental Therapeutics, [5]Center for Individualized Medicine, Mayo Clinic College of Medicine and Science, Mayo Clinic, 200 First Street SW, Rochester, MN 55905

*Corresponding Author: Hu Li, Ph.D.

Email: li.hu@mayo.edu

Telephone: 507-293-1182

Supplementary Table S1. Annotated nodes in prioritized subnetwork.

Node	Name	Description
**AHSA1**	Activator of 90 kDa heat shock protein ATPase homolog 1	acts as a co-chaperone of HSP90AA1. Activates the ATPase activity of HSP90AA1
**CASP3**	Caspase-3	
**CCL5**	Chemokine (C-C motif) ligand 5	chemotactic for T cells, eosinophils, and basophils, and plays an active role in recruiting leukocytes into inflammatory sites
**CFTR**	Cystic fibrosis transmembrane conductance regulator	
**COPS5**	COP9 constitutive photomorphogenic homolog subunit 5	regulator of signaling pathways, reported to be involved in the degradation of cyclin-dependent kinase inhibitor CDKN1B/p27Kip1
**CUL3**	Cullin-3	core piece of E3 ubiquitin ligase complex
**DCP2**	mRNA-decapping enzyme 2	key component of an mRNA-decapping complex required for removal of the 5-prime cap from mRNA prior to its degradation from the 5-prime end
**DDX58**	retinoic acid-inducible gene I	RIG-I-like receptor dsRNA helicase enzyme
**FN1**	Fibronectin	glycoprotein of the extracellular matrix that binds to integrin
**GARS**	glycyl-tRNA synthetase i	one of the aminoacyl-tRNA synthetases that charge tRNAs with their cognate amino acids
**GTPBP1**	GTP-binding protein 1	GTP-binding protein
**HLA-E**	HLA class I histocompatibility antigen, alpha chain E	cell recognition by natural killer cells
**HNRNPA1**	Heterogeneous nuclear ribonucleoprotein A1	are associated with pre-mRNAs in the nucleus and appear to influence pre-mRNA processing and other aspects of mRNA metabolism and transport
**HSP90AA1**	Heat shock protein HSP 90-alpha	initiated when a cell experiences proteotoxic stress
**HUWE1**	HECT, UBA and WWE domain containing 1, E3 ubiquitin protein ligase	regulates apoptosis by catalyzing the polyubiquitination and degradation of MCL1
**IFIT1**	Interferon-induced protein with tetratricopeptide repeats 1	may inhibit viral replication and translational initiation
**IFIT3**	Interferon-induced protein with tetratricopeptide repeats 3	acts as an inhibitor of cellular as well as viral processes, cell migration, proliferation, signaling, and viral replication
**IKBKE**	Inhibitor of nuclear factor kappa-B kinase subunit epsilon	regulating inflammatory responses to viral infection, through the activation of the type I IFN, NF-kappa-B and STAT signaling
**IL32**	Interleukin 32	pro-inflammatory cytokine that can induce cells of the immune system (such as monocytes and macrophages) to secrete inflammatory cytokines, such as tumor necrosis factor-alpha (TNF-α) and IL-6. In addition, it can also induce the production of chemokines such as IL-8 and MIP-2 / CXCL2.
**IMPDH2**	Inosine-5'-monophosphate dehydrogenase 2	rate-limiting enzyme in the de novo guaninenucleotide biosynthesis

**IRF7**	Interferon regulatory factor 7	Play a role in the transcriptional activation of virus-inducible cellular genes, including the type I interferon genes. In particular, IRF7 regulates many interferon-alpha genes. Constitutive expression of IRF7 is largely restricted to lymphoid tissue, largely plasmacytoid dendritic cells, whereas IRF7 is inducible in many tissues. Multiple IRF7 transcript variants have been identified, although the functional consequences of these have not yet been established.
**LYN**	Tyrosine-protein kinase Lyn	inhibitory role in myeloid lineage proliferation
**MCM3**	DNA replication licensing factor MCM3	one of the highly conserved mini-chromosome maintenance proteins (MCM) that are involved in the initiation of eukaryotic genome replication
**MCM7**	DNA replication licensing factor MCM7	one of the highly conserved mini-chromosome maintenance proteins (MCM) that are involved in the initiation of eukaryotic genome replication
**NCL**	Nucleolin	a eukaryotic nucleolar phosphoprotein, is involved in the synthesis and maturation of ribosomes
**P4HA1**	Prolyl 4-hydroxylase subunit alpha-1	key enzyme in collagen synthesis
**PAN2**	PAN2 poly(A) specific ribonuclease subunit	deadenylase that functions as the catalytic subunit of the polyadenylate binding protein dependent poly(A) nuclease complex
**PF4**	Platelet factor 4	70-amino acid protein that is released from the alpha-granules of activated platelets and binds with high affinity to heparin (to neutralize heparin)
**PIGR**	Polymeric immunoglobulin receptor	Fc receptor which facilitates the secretion of the soluble polymeric isoforms of immunoglobulin A and immunoglobulin M (expressed on several glandular epithelia including those of liver and breast)
**PLAT**	Plasminogen activator, tissue type	a secreted serine protease that converts the proenzyme plasminogen to plasmin, a fibrinolytic enzyme
**POLR2H**	RNA polymerase II subunit H	one of the essential subunits of RNA polymerase II that is shared by the other two eukaryotic DNA-directed RNA polymerases, I and III
**PRKAR1A**	Protein Kinase CAMP-Dependent Type I Regulatory Subunit Alpha	PRKAR1A leads to the Carney complex, associating multiple endocrine tumors
**PRKCB**	Protein Kinase C Beta	B cell activation, apoptosis induction, endothelial cell proliferation, and intestinal sugar absorption
**RPA3**	Replication protein A 14 kDa subunit	interact with replication protein A1[5][6] and replication protein A2
**RPL6**	60S ribosomal protein L6	may participate in tax-mediated transactivation of transcription
**SERPINA5**	serpin family A member 5	glycoprotein that can inhibit several serine proteases, including protein C and various plasminogen activators and kallikreins, and it thus plays diverse roles in hemostasis and thrombosis in multiple organs
**SKIL**	SKI like proto-oncogene	component of the SMAD pathway, which regulates cell growth and differentiation through transforming growth factor-beta (TGFB)

**SMAD4**	Mothers against decapentaplegic homolog 4	Mutations or deletions in this gene have been shown to result in pancreatic cancer, juvenile polyposis syndrome, and hereditary hemorrhagic telangiectasia syndrome.
**SMAD6**	Mothers against decapentaplegic homolog 6	Heterozygous, damaging mutations in SMAD6 are the most frequent genetic cause of non-syndromic craniosynostosis identified to date
**SPTBN1**	Spectrin beta chain, brain 1	functions in the determination of cell shape, arrangement of transmembrane proteins, and organization of organelles
**STAT1**	Signal transducer and activator of transcription 1	mediates the expression of a variety of genes, which is thought to be important for cell viability in response to different cell stimuli and pathogens
**STAT2**	Signal transducer and activator of transcription 2	forms a complex with STAT1 and IFN regulatory factor family protein p48 (IRF9), in which this protein acts as a transactivator, but lacks the ability to bind DNA directly.
**STIP1**	Stress induced phosphoprotein 1	coordinates the functions of HSP70 (see HSPA1A; MIM 140550) and HSP90 (see HSP90AA1; MIM 140571) in protein folding. It is thought to assist in the transfer of proteins from HSP70 to HSP90 by binding both HSP90 and substrate-bound HSP70. STIP1 also stimulates the ATPase activity of HSP70 and inhibits the ATPase activity of HSP90, suggesting that it regulates both the conformations and ATPase cycles of these chaperones
**SUMO1**	Small ubiquitin-related modifier 1	a member of the SUMO (small ubiquitin-like modifier) protein family. It functions in a manner similar to ubiquitin in that it is bound to target proteins as part of a post-translational modification system. However, unlike ubiquitin which targets proteins for degradation, this protein is involved in a variety of cellular processes, such as nuclear transport, transcriptional regulation, apoptosis, and protein stability.
**THBD**	Thrombomodulin	integral membrane protein expressed on the surface of endothelial cells and serves as a cofactor for thrombin. It reduces blood coagulation by converting thrombin to an anticoagulant enzyme from a procoagulant enzyme
**TUT1**	Terminal uridylyl transferase 1, U6 snRNA-specific	adds and removes nucleotides from the 3' end of small nuclear RNAs and select mRNAs and may function in controlling gene expression and cell proliferation.
**UBC**	Ubiquitin C	protein degradation, DNA repair, cell cycle regulation, kinase modification, endocytosis, and regulation of other cell signaling pathways.
**XRN2**	5'-3' Exoribonuclease 2	involved in the torpedo model of transcription termination
**YBX1**	Y box binding protein 1	functions as both a DNA and RNA binding protein and has been implicated in numerous cellular processes including regulation of transcription and translation, pre-mRNA splicing, DNA reparation and mRNA packaging
**ZC3HAV1**	Zinc finger CCCH-type antiviral protein 1	CCCH-type zinc finger protein that is thought to prevent infection by retroviruses. Studies of the rat homolog indicate that the protein may primarily function to inhibit viral gene expression and induce an innate immunity to viral infection.

Supplementary Table S2. GBM cell lines used in gene dependency analyses. Cell lines are listed by Broad Institute DepMap ID.

GBM cell line	
ACH-000283	ACH-000215
ACH-000558	ACH-002268
ACH-000269	ACH-000455
ACH-001329	ACH-000673
ACH-000464	ACH-000676
ACH-002223	ACH-000760
ACH-002224	ACH-000152
ACH-002225	ACH-000200
ACH-002226	ACH-001605
ACH-002227	ACH-001606
ACH-002228	ACH-001608
ACH-002229	ACH-001609
ACH-002230	ACH-001610
ACH-002231	ACH-001611
ACH-000863	ACH-001622
ACH-000244	ACH-001623
ACH-000098	ACH-001624
ACH-000738	ACH-000887
ACH-000756	ACH-000376
ACH-000102	ACH-001198
ACH-000027	ACH-000368
ACH-000231	ACH-000623
ACH-000622	ACH-000289
ACH-000445	ACH-000543
ACH-000479	ACH-000370
ACH-002259	ACH-000571
ACH-000819	ACH-000208
ACH-000328	ACH-000036
ACH-000595	ACH-000469
ACH-000634	ACH-000570

Supplementary Figure S1. KEGG pathway for HSV-1 infection (hsa05168; https://www.kegg.jp/)[1] with overrepresented nodes from LN229 context specific network annotated (red).

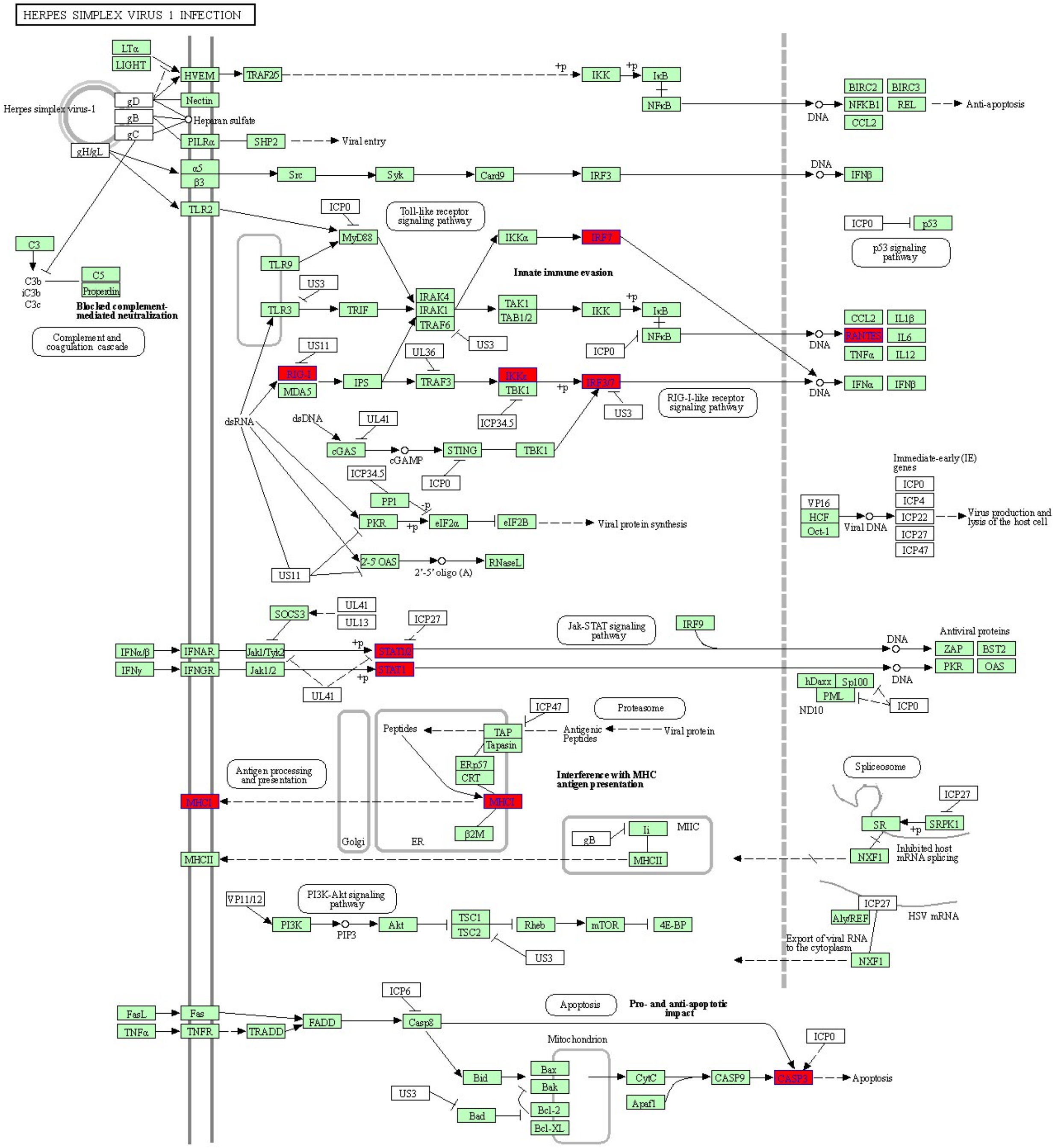

Supplementary Figure S2. (**a**) CCN1 expression and correlation with IDH1 (ρ = -0.273; p value = 0.027). (**b**) Box plot of CCN1 expression in all CCLE GBM cell lines (n = 66) median expression value was used to classify cell lines in CCN1-high and CCN1-low for downstream NetDecoder analyses.

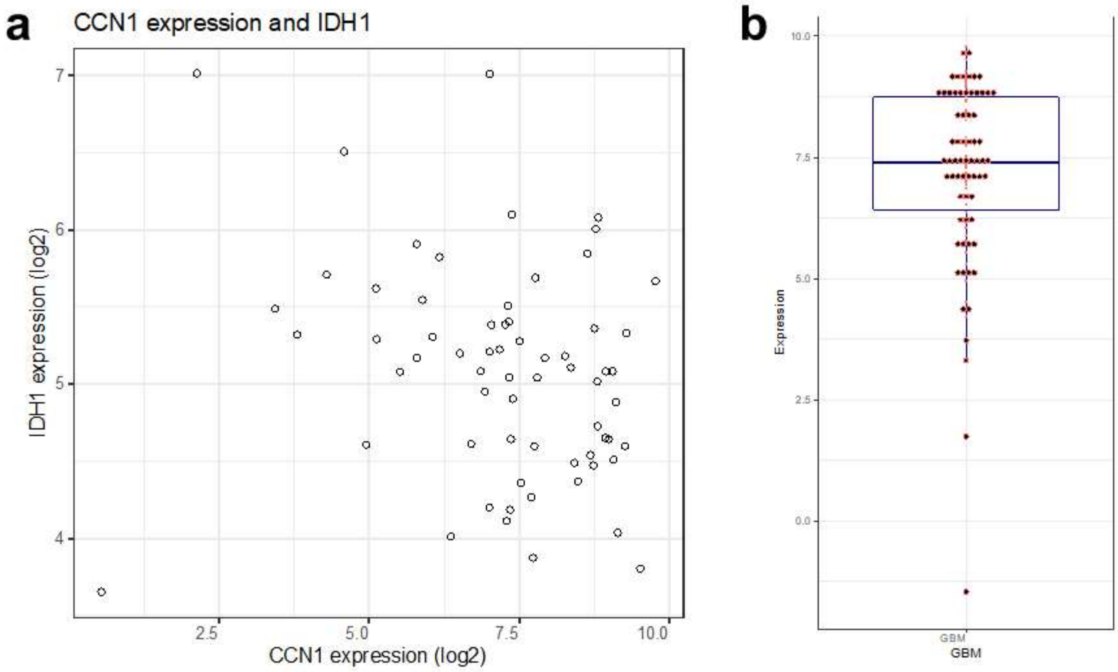

Supplementary Figure S3. NetDecoder analysis of CCLE and TCGA GBMs.

Prioritized subnetwork of (**a**) CCLE GBMs using the published LN229 sources and (**b**) TCGA GBMs using the published LN229 sources. (**c**) Venn diagram identifies common prioritized subnetwork genes across CCLE and TCGA datasets.

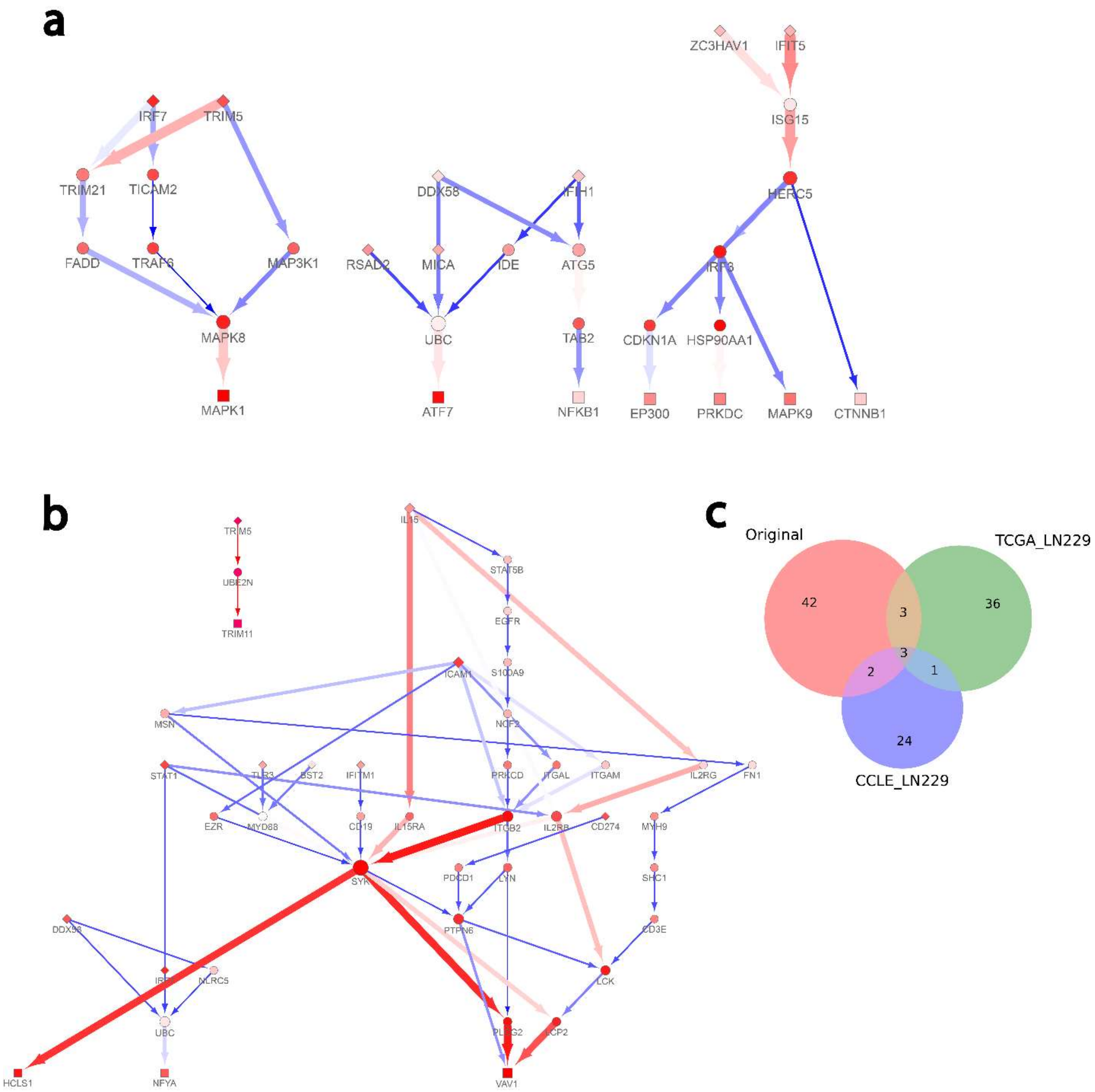

References

1 Kanehisa, M., Furumichi, M., Sato, Y., Ishiguro-Watanabe, M. & Tanabe, M. KEGG: integrating viruses and cellular organisms. *Nucleic Acids Res* **49**, D545-D551, doi:10.1093/nar/gkaa970 (2021).
